# Functional expression and activity of the recombinant antifungal defensin *Pv*D_1_r from *Phaseolus vulgaris* L. (common bean) seeds

**DOI:** 10.1186/1471-2091-15-7

**Published:** 2014-04-01

**Authors:** Érica de O Mello, Izabela S dos Santos, André de O Carvalho, Luísa S de Souza, Gonçalo A de Souza-Filho, Viviane V do Nascimento, Olga LT Machado, Umberto Zottich, Valdirene M Gomes

**Affiliations:** 1 Laboratório de Fisiologia e Bioquímica de Microrganismos do Centro de Biociências e Biotecnologia da Universidade Estadual do Norte Fluminense Darcy Ribeiro, Avenida Alberto Lamego 2000, Campos dos Goytacazes, RJ, Brazil; 2 Universidade Estadual do Norte Fluminense Darcy Ribeiro, Laboratório de Biotecnologia, Campos dos Goytacazes 28013-602RJ, Brazil; 3 Universidade Estadual do Norte Fluminense Darcy Ribeiro, Laboratório de Química e Função de Proteínas e Peptídeos, Campos dos Goytacazes 28013-602RJ, Brazil; 4 Universidade de Vila Velha, Laboratório de Microbiologia Ambiental e Biotecnologia, 28050620, Vila Velha, ES, Brazil

**Keywords:** Antimicrobial activity, Antimicrobial peptide, Common bean, Functional expression, Plant defensin

## Abstract

**Background:**

Defensins are basic, cysteine-rich antimicrobial peptides that are important components of plant defense against pathogens. Previously, we isolated a defensin, *Pv*D_1_, from *Phaseolus vulgaris* L. (common bean) seeds.

**Results:**

The aim of this study was to overexpress *Pv*D_1_ in a prokaryotic system, verify the biologic function of recombinant *Pv*D_1_ (*Pv*D_1_r) by comparing the antimicrobial activity of *Pv*D_1_r to that of the natural defensin, *Pv*D_1_, and use a mutant *Candida albicans* strain that lacks the gene for sphingolipid biosynthesis to unravel the target site of the *Pv*D_1_r in *C. albicans* cells. The cDNA encoding *Pv*D_1_, which was previously obtained, was cloned into the pET-32 EK/LIC vector, and the resulting construct was used to transform bacterial cells (Rosetta Gami 2 (DE_3_) pLysS) leading to recombinant protein expression. After expression had been induced, *Pv*D_1_r was purified, cleaved with enterokinase and repurified by chromatographic steps. N-terminal amino acid sequencing showed that the overall process of the recombinant production of *Pv*D_1_r, including cleavage with the enterokinase, was successful. Additionally, modeling revealed that *Pv*D_1_r had a structure that was similar to the defensin isolated from plants. Purified *Pv*D_1_ and *Pv*D_1_r possessed inhibitory activity against the growth of the wild-type pathogenic yeast strain *C. albicans*. Both defensins, however, did not present inhibitory activity against the mutant strain of *C. albicans*. Antifungal assays with the wild-type *C. albicans* strains showed morphological changes upon observation by light microscopy following growth assays. *Pv*D_1_r was coupled to FITC, and the subsequent treatment of wild type *C. albicans* with DAPI revealed that the labeled peptide was intracellularly localized. In the mutant strain, no intracellular labeling was detected.

**Conclusion:**

Our results indicate that *Pv*D_1_r retains full biological activity after recombinant production, enterokinase cleavage and purification. Additionally, our results from the antimicrobial assay, the microscopic analysis and the *Pv*D_1_r-FITC labeling assays corroborate each other and lead us to suggest that the target of P*v*D_1_ in *C. albicans* cells is the sphingolipid glucosylceramide.

## Background

Plant defensins are cationic peptides that are ubiquitous in the plant kingdom and belong to a large superfamily of antimicrobial peptides that are found in several organisms [1]. The plant defensins protein family is composed of small peptides consisting of 45–54 amino acids and are encompassed in a three-dimensional structure containing three anti-parallel β-strands and one α-helix [2,3]. The three-dimensional structure is stabilized by four disulfide bridges formed by eight strictly conserved cysteine residues. Two of these bridges compose the cysteine-stabilized α-helix β-strand motif that is also found in other peptides with similar biological activities [4,5].

The antimicrobial activity of plant defensins was first reported in the early 1990s by Terras et al. [1]. The antimicrobial activity of plant defensins has been observed mainly against fungi; however, some bacteria, especially Gram-positive species, have also been shown to be inhibited, although the activity of defensins against bacteria is less pronounced than the activity against fungi. The growth of several fungal species, including several filamentous fungi and yeasts, is inhibited when incubated with defensins [6-9]. Based on their antifungal properties, plant defensins are classified into two groups: group I includes plant defensins that inhibit hyphal elongation with morphological changes and group II consists of those that inhibit fungal growth without morphological changes [3]. The antifungal activity of plant defensins appears to require specific binding targets on fungal membranes [10]. The molecular mechanism underlying the antimicrobial mode of action is still poorly understood, although specific targets on fungal membranes have been identified for the defensins from *Raphanus sativus* L. and *Dahlia merckii*[10,6].

*Phaseolus vulgaris* L. was originally cultivated in the New World but is now grown extensively in all major continental areas [11]. It is a principal dietary component and protein source for more than 300 million people in Latin America and Western and South Africa [12,13]. Brazil is the largest producer of common beans, and the cultivation of this crop is socially important because it is farmed as a subsistence crop and is the main source of income for small farmers [7].

Previously, Games et al. [7] isolated and characterized a plant defensin from the seeds of *P. vulgaris* named *Pv*D_1_ (*Phaseolus vulgaris* defensin one), and this defensin exhibited antifungal activity against different yeast strains. This same study also reported the cloning of *Pv*D_1_ cDNA by RT-PCR. In the present study, we continue to explore the function of *Pv*D_1_ by demonstrating the functional expression and antimicrobial activity of the recombinant form of *Pv*D_1_ (*Pv*D_1_r). Additionally, through the use of a mutant yeast strain deficient in sphingolipid synthesis, we begin to elucidate the membrane target of *Pv*D_1_.

## Methods

### Materials

*Phaseolus vulgaris* L. seeds were supplied by the Empresa de Pesquisa Agropecuária do Estado do Rio de Janeiro – Pesagro, Campos dos Goytacazes, RJ, Brazil.

The cDNA encoding the defensin *Pv*D_1_ was obtained as described by [7].

Chemically competent cells of the *Escherichia coli* bacterial strains JM 109 [genotype: *end*A1, *rec*A1, *gyr*A96, *thi*, *hsd*R17, (r_k_^-^, m_k_^+^), *rel*A1, *sup*E44, λ^-^, Δ(*lac*-*pro*AB), [F’, *tra*D36, *pro*AB, *lac*I^q^ZΔM15]] (Promega Corporation, USA) and Rosetta-gami 2 (DE_3_) pLysS [genotype: Δ(*ara*–*leu*)*7697* Δ*lacX74* Δ*phoA PvuII phoR araD139 ahpC galE galK rpsL* (DE_3_) F’[*lac*^+^*lacI*^q^*pro*] *gor522*::Tn*10 trxB* pLysSRARE23 (Cam^R^, Str^R^, Tet^R^)^4^)] (Novagen) were used. Luria-Bertani (LB) medium was used as a routine bacterial growth and expression medium.

*Candida albicans* mutant strain (Δ)GCS1, deficient on glucosyl ceramide synthase (GCS) enzyme, was kindly provided by Dr. Dirk Warnecke from the Institut fur Allgemeine Botanik, University of Hamburg, Germany, and was obtained from the Instituto de Biofísica Carlos Chagas Filho, Universidade Federal do Rio de Janeiro, Rio de Janeiro, Brazil. Yeast cultures were maintained on potato dextrose agar (potato infusion 0.4%, dextrose 2.0%, agar 1.5%).

### Extraction and purification of the *Pv*D_1_ from *P. vulgaris* seeds

The natural defensin from *P. vulgaris* seeds, *Pv*D_1_, was purified as follows. Fine flour (100 g) was prepared from the seeds of *P. vulgaris* in a mill. A protein extract was prepared from this flour using 500 mL of extraction buffer (10 mM Na_2_HPO_4_, 15 mM NaH_2_PO_4_, 100 mM KCl, 1.5% EDTA, pH 5.4) for 2 h at 4°C with constant agitation. This protein extract was centrifuged at 15,000 *g*, and the supernatant was fractionated at a 70% relative ammonium sulfate saturation at 4°C for 18 h. After centrifugation under the same conditions, the precipitate was redissolved in distilled water and heated at 80°C for 15 min in a water bath. This heated protein extract was centrifuged at 10,000 *g*. The supernatant was recovered and extensively dialyzed against distilled water for three days and then recovered by freeze drying. For peptides, purification was initially performed on a DEAE-Sepharose column (with 100 mL of resin) equilibrated with 20 mM Tris–HCl (pH 8.0) at flow rate of 60 mL/h. The freeze dried protein extract (50 mg) was reconstituted in 5 mL of the equilibrium buffer and centrifuged (16,000 *g*, 3 min at 4°C), and the supernatant was loaded onto the column. A non-retained fraction (D1) was eluted in the equilibrium buffer, and a bound fraction (D2) was eluted in the same buffer with 1 M NaCl. The D1 fraction was freeze dried to concentrate, resuspended in 0.1% (v/v) trifluoroacetic acid (TFA) and injected into an HPLC C2C18 reversed-phase column (μRPC C2/C18 ST 4.6/100, GE Healthcare) attached to a C8 guard column (Pelliguard, Sigma). The bound peptides were eluted with an acetonitrile gradient starting with 100% solvent A (0.1% TFA) for 10 min followed by a mixture of solvent A and solvent B (80% acetonitrile containing 0.1% TFA) from 0 to 100% for 48 min and was then washed with 100% solvent B for 10 min. The concentration of solvent A rose to 100% following the 60 min total chromatography run, as shown by Games et al. [1].

### Construction of the recombinant expression vector

The strategic design for the expression vector was based on the amplification of the cDNA encoding *Pv*D_1_ using a specific set of primers that were designed to allow the amplicon to be cloned into a pET-32 EK/LIC expression vector (Novagen). This vector is part of a system for the expression of recombinant proteins that are fused to the thioredoxin (Trx tag), which facilitates protein solubility. Additionally, this vector provides six consecutive histidines (His) and glutathione (S) tags for the purification and identification of recombinant proteins. After purification, all tags can be completely removed via a cleavage reaction with the endoproteinase enterokinase (EK), and the proteolytic site is provided by the vector.

### Fragment preparation for cloning into the expression vector

One set of primers was designed for the cloning of *Pv*D_1_ into the pET-32 EK/LIC vector, according to the manufacturer’s instruction. The primers were as follows: 5′-*GACGACGACAAG*ATG**AAGACGTGCGAGAACCTG**-3′ for the sense primer (Ds) and 5′-*GAGGAGAAGCCCGGT***TTAACAGTTTTTGGT**-3′ for the antisense primer (Das). The bold letters correspond to the sequence that anneals to the coding sequence of the *Pv*D1 cDNA, and the underlined letters correspond to the sequence that anneals to the vector and were introduced to generate specific overhangs that would anneal with the pET-32 EK/LIC vector after treatment with T_4_ DNA polymerase. The double waves indicate a methionine codon, which was included according to the instructions furnished by the manufacturer (the sequence coding for the defensin does not include this codon naturally). This strategy permits directional cloning without the need for restriction digestion or ligation by a process called ligation-independent cloning (LIC). The primers were synthesized by Invitrogen, USA/Life Technologies, Brazil.

PCRs for fragment amplification were performed with a Mastercycler gradient 22331 (Eppendorf), and the PCR mixture contained 1× Fideli*Taq* buffer (GE Healthcare), 0.2 mM dNTPs, 20 μM Ds, 20 μM Das, 1 μL of cDNA, and 0.5 units of Fideli*Taq* DNA polymerase I (GE Healthcare) in a final volume of 20 μL per reaction. The reactions were initially warmed at 95°C for 1.5 min, followed by 45.3°C for 45 s, 68°C for 1.5 min, 35 cycles of the following program: 95°C for 45 s, 58.7°C for 45 s, and 68°C for 1.5 min.

The PCR products were directly purified with Wizard SV gel and a PCR clean-up system (Promega), according to the manufacturer’s instructions, and were treated with T_4_ DNA polymerase to generate overhang ends that were compatible with the vector. The T4 DNA polymerase reactions contained the following reagents: 300 ng of the purified fragment, 1× T_4_ DNA polymerase buffer (33 mM Tris-acetate (pH 7.9), 66 mM sodium acetate, 10 mM magnesium acetate, 1 mM DTT), 2.5 mM dATP, 5 mM DTT, and 5 units of T_4_ DNA polymerase in a 20 μL reaction. The reactions were incubated at 22°C for 30 min, and subsequently, the T_4_ DNA polymerase was inactivated by incubating the reactions at 75°C for 20 min.

### pET-32 EK/LIC preparation and annealing

The pET-32 EK/LIC vector was furnished by the manufacturer and annealed to the fragment encoding the defensin, which was prepared as described above. The annealing reaction consisted of 50 ng of pET-32 EK/LIC and 2 μL of the T_4_ DNA polymerase-treated fragment. The reaction was incubated at 22°C for 5 min, and 1 μL of 25 mM EDTA was subsequently added, followed by a second incubation at 22°C for 5 min. From this reaction, 1 μL was used for bacterial (*E. coli* JM 109) transformation.

### Transformation and colony screening

The transformation of JM 109 competent cells was performed as described by Inoue et al. [14], and the screening was performed by a plasmid extraction and digestion (*Eco* RI and *Bgl* II). The resulting DNA construct was named pET-*Pv*D_1_. After successful cloning was confirmed, the construct pET-*Pv*D_1_ was used to transform the super-expression strain of *E. coli* Rosetta-gami 2 (DE_3_) pLysS competent cells, according to the manufacturer’s instructions. Screening was performed by PCR directly from the bacterial colonies as follows: three colonies were randomly selected from the agar plate using sterile pipette tips, transferred to PCR tubes that contained 10 μL of sterile water, and homogenated with the pipette tips. Ten microliters of a mixture that contained 1× *Taq* buffer (GE Healthcare), 0.2 mM dNTPs, 20 μM Ds, 20 μM Das, and 0.5 units Fideli*Taq* DNA polymerase I (GE Healthcare) were added to these tubes to yield a final volume of 20 μL. These reactions were submitted for PCR analysis. The PCR products were loaded onto a 1% agarose gel.

### *Pv*D_1_r expression and purification

The transformed cells were grown at 37°C in liquid LB medium (0.5% yeast extract, 1.0% tryptone, 1.0% NaCl) supplemented with ampicillin (50 μg.mL^-1^) and chloramphenicol (34 μg.mL^-1^) to an optical density (at 600 nm) of 0.5 before induction with 1 mM IPTG. After 3 h of induction, the cells were harvested by centrifugation (4,000 *g* for 15 min at 4°C); suspended in 50 mM phosphate buffer (pH 7.4) containing 500 mM NaCl, 40 mM imidazole, 1 mM PMSF, and a protease inhibitor cocktail for general use (Sigma, USA), according to the manufacturer’s manual; and ruptured by sonication (10 pulses of 30 sec at 10 watts) (R2D91109, Unique). Next, the cell lysate was clarified by centrifugation (5,000 *g* for 10 min at 4°C) (5430R, Eppendorf), and the supernatant was heated at 90°C for 30 min. The resulting suspension was centrifuged (7,200 *g* for 10 min at 4°C), and the supernatant was purified further by chromatographic methods.

Initially, Ni^+^-NTA agarose resin (Qiagen) was used to purify the soluble fusion protein by affinity chromatography. The Ni^+^-NTA agarose resin was equilibrated with a 50 mM phosphate buffer (pH 7.4) that contained 500 mM NaCl and 40 mM imidazole. The bound fraction was eluted with 500 mM imidazole in a 50 mM phosphate buffer (pH 7.4). To separate *Pv*D_1_r from the tags (Trx, S and His) provided by the vector, cleavage of the fusion protein was performed by incubating the bound fraction with recombinant bovine enterokinase (Sigma, Co.) for 16 h at 25°C, according to the manufacturer’s instruction. After cleavage, this fraction was resubmitted for affinity chromatography on Ni^+^-NTA agarose resin under the conditions described above. At this point, the recombinant defensin had lost affinity to the Ni^+^ because it did not have the His tag and was therefore found in the unbounded fraction. This purified *Pv*D_1_r was submitted to the C2C18 reversed-phase column exactly as described in the purification of the natural *Pv*D_1_ and the retention time was recorded. In the second round of purification of the *Pv*D_1_r, the cleavage sample was applied directly into the C2C18 reversed-phase column, and the peak with the same retention time of the *Pv*D_1_r, as previously recorded, was collected.

The purification process was monitored by SDS-tricine gel electrophoresis performed according to the method of Schägger and von Jagow [15].

### *Pv*D_1_r amino acid sequencing

The purified *Pv*D_1_r that was obtained after Ni^+^ affinity chromatography underwent Edman automated N-terminal amino acid sequencing [16] on a Shimadzu PPSQ-10 Automated Protein Sequencer. The sequence was determined from the purified peptide that was blotted onto PVDF membranes after SDS-tricine gel electrophoresis. PTH-amino acids were detected at 269 nm after separation on a reverse-phase C4 column (4.6 × 2.5 mm) under isocratic conditions, according to the manufacturer’s instructions. The sequences were compared to sequences reported in amino acid data banks and were submitted for automatic alignment using the NCBI-BLAST search system.

### Modeling

The three-dimensional structure of *Pv*D_1_r was modeled with the Modeller Program [17]. Initially, a search of a protein sequence databank was performed using the sequence of the *Pv*D_1_r as a query in BLASTP [18]. Based on this analysis, the *Vigna radiata* defensin 2 (VrD2; pdb CODE 2GL1) was selected as the template. The *Pv*D_1_r model was generated, and minimization of energy (approximately 10) was performed to optimize the geometric parameters of the model. The minimization of the model was achieved using the Gromos96 Swiss-PDB Viewer program. The stereochemical quality of the models was checked using the Ramachandran plots (PROCHECK program) and Profile 3D, both of which were available on the PARMODEL web server [19,20], and several parameters were analyzed, including the peptide bonds, planarity of the rings of the side chains, and twist angles ϕ and ψ of the main chain.

### *Candida albicans* growth inhibition assay

For the preparation of the yeast cell cultures, an inoculum was transferred to Petri dishes that contained potato dextrose agar, and the inoculates were allowed to grow on the plates at 30°C for 2 days. Next, the cells were transferred to sterile culture medium (10 mL). The yeast cells were quantified in a Neubauer chamber for further calculations of appropriate dilutions. A quantitative assay for yeast growth inhibition was performed according to the protocol developed previously by Broekaert et al. [21], with some modifications. To monitor the effects of *Pv*D_1_ and *Pv*D_1_r on the growth of fungal cells, the cells were incubated in microplates at 30°C, at a final volume of 200 μL (10.000 cells in 1 mL of potato dextrose broth), and in the presence or absence (general control) of *Pv*D_1_ and *Pv*D_1_r (100 μg.mL^-1^). Optical readings at 620 nm were taken at timepoint zero and at every 6 h for the following 24 h. The readings were taken against a blank that contained only the culture medium. All of the experiments were run in triplicate, and the reading averages, standard errors and coefficients of variation were calculated.

### Optical microscopy analysis and localization of *Pv*D_1_r conjugated to FITC in *Candida albicans* cells

For optical microscopy analysis, after a 24 h yeast growth inhibition assay with *Pv*D_1_ and *Pv*D_1_r, the yeast cells were separated from the growth medium by centrifugation (4,000 *g* for 5 min at 4°C), washed in potato dextrose broth and visualized with an optical microscope (Axiovert 135, Zeiss). The yeast cells that were grown in the absence of defensins were also analyzed.

For localization analysis, *Pv*D_1_r was conjugated to fluorescein isothiocyanate (FITC), according to the manufacturer’s instructions for FITC (Sigma). The nuclei were stained with 50 μg.mL^-1^ of 4’,6-diamidino-2-phenylindole dihydrochloride (DAPI) for 10 min, which was followed by fluorescence analysis with image analysis software (Axiovision®) and an Axioplan Zeiss microscope.

## Results

### Protein expression and purification

**Figure 1:**
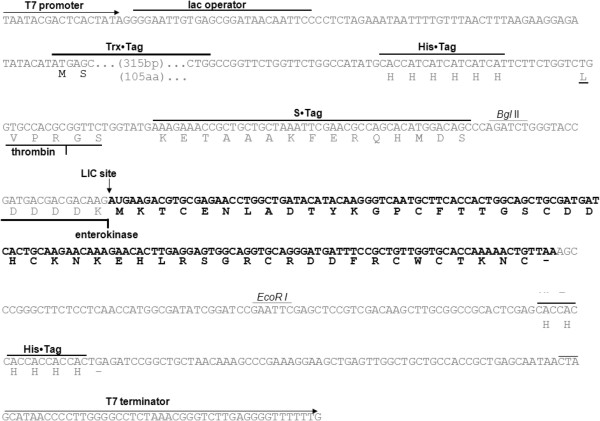
**Sequence map of the cloning of *Pv*D**_**1 **_**into the expression vector pET-32 EK/LIC.** The coding sequence of the *Pv*D_1_ (in bold) is shown linked to the LIC site of the vector pET-32 EK/LIC (indicated by a vertical arrow). The T7 promoter and terminator (horizontal arrows), lac operator, thioredoxin (Trx), six consecutive histidines (His) and (S) cleavable tags, thrombin and enterokinase protease sites, and His tag and terminator codon (-) are shown on the sequence map. The relevant amino acid sequences are shown below the nucleotide sequence. After expression and purification of the recombinant protein in this vector, all of the tags can be separated from the recombinant *Pv*D_1_ by a combined treatment with enterokinase as well as another chromatographic step.

**Figure 2:**
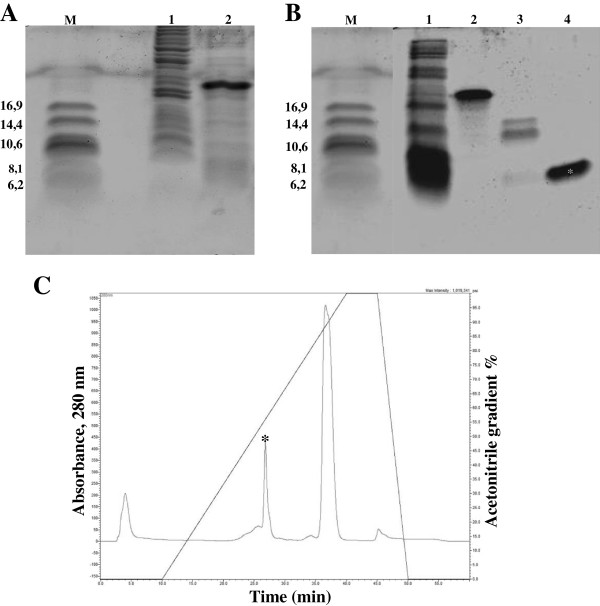
**SDS-tricine gel electrophoresis analysis of *Pv*D**_**1**_**r expression and purification. (A)** Lane 1, protein extract of the uninduced pET-*Pv*D_1_ transformed Rosetta-gami 2 bacteria; lane 2, protein extract of the induced pET-*Pv*D_1_ transformed Rosetta-gami 2 bacteria. **(B)** Lane 1, N1 peak obtained from Ni^+^ affinity chromatography; lane 2**,** N2 peak obtained from Ni^+^ affinity chromatography; lane 3, N2 peak after enterokinase cleavage; lane 4**,** Purified *Pv*D_1_r obtained from the C2C18 reversed-phase column; M - low molecular mass marker (kDa). **(C)** Chromatogram of the last step of the purification of *Pv*D_1_r after cleavage of N2 with enterokinase. The oblique line indicates the acetonitrile gradient. The retention time of *Pv*D_1_r was previously determined by purified *Pv*D_1_r in Ni^+^-NTA agarose. The same retention time was collected and this sample presented only one band by tricine gel electrophoresis (Figure 2B4). The peak and the corresponding band are indicated by asterisks.

The coding sequence of the mature *P. vulgaris* seed defensin, obtained via PCR amplification [7], was inserted into the pET-32 EK/LIC expression vector (Figure 1). The resulting construct, named pET-*Pv*D_1_, was subsequently transformed into the *E. coli* JM 109 strain. Positive colonies were screened by plasmid extraction and digestion, and the construct pET-*Pv*D_1_ was used to transform the overexpressing *E. coli* strain Rosetta-gami 2 (DE_3_) pLysS. Following an induction by IPTG, the strain transformed with pET-*Pv*D_1_ expressed, as confirmed by SDS-tricine gel electrophoresis, a fusion protein with a molecular mass above the approximately 16.9 kDa marker that corresponded to the *Pv*D_1_r fused with both the Trx and His tags (referred to as Trx-His-*Pv*D_1_r) (Figure 2A2). After induction, the soluble cell extract, containing Trx-His-*Pv*D_1_r, was initially fractionated via metal affinity chromatography on a Ni^+^-NTA agarose column and two different peaks were observed (result not shown). The first was unbound to the resin and corresponded to the fractions that do not present affinity to the Ni^+^ (N1), and the second bound to the resin (N2) and presented proteins that had an affinity to the Ni^+^ (result not shown). The bound fraction, as analyzed by SDS-tricine gel electrophoresis, contained *Pv*D_1_r bound to Ni^+^ by the His tag, (Figure 2B2). In this same lane, low molecular mass contaminants that also present affinity to Ni^+^ were observed (Figure 2B2). The bound fraction (N2) containing Trx-His-*Pv*D_1_r was submitted to treatment with the recombinant bovine enterokinase to split the Trx-His tags from *Pv*D_1_r (result not shown). Analysis by SDS-tricine gel electrophoresis showed that after the enterokinase treatment, the band above the 16.9 kDa marker disappeared and a protein band of approximately 6 kDa appeared (Figure 2B3). The enterokinase treated sample was subsequently recovered and resubmitted to the Ni^+^-NTA agarose column, as described above, to achieve a final purification of *Pv*D_1_r. Once again, two peaks were obtained, one unbound and one bound (result not shown). Here the unbound fraction corresponded to the *Pv*D_1_r that had lost affinity to the Ni^+^ because it was no longer fused with the His tag (result not shown). For the final purification step of *Pv*D_1_r and also to confirm its purity, the cleaved sample was submitted for chromatography on a C2C18 reversed-phase column. Purified *Pv*D_1_r was passed on this column by second metal affinity chromatography, and the retention time of *Pv*D_1_r was recorded. The chromatogram presented two main peaks, and the peak corresponding to the retention time of the *Pv*D_1_r was collected (Figure 2C, indicated by an asterisk). SDS-tricine gel electrophoresis showed only one band of approximately 6 kDa (Figure 2B4, indicated by an asterisk).

### Amino acid sequencing

The 6 kDa peptide underwent automated N-terminal amino acid sequencing, and the analysis of its eight N-terminal amino acids, counted after the first methionine, matched exactly the first eight N-terminal amino acid sequence of the defensin from *P. vulgaris* seeds, *Pv*D_1_, as previously described by Games et al. [7]. The obtained partial N-terminal sequence and the first eight N-terminal sequences of *Pv*D_1_ differed in only one amino acid residue, an initial methionine, which was added intentionally as a requirement for protein expression via the pET-32 EK/LIC system. The generated partial N-terminal sequence of the 6 kDa peptide clearly demonstrated that the overall process of recombinant production of *Pv*D_1_r, including the cleavage with the enterokinase, was successful (result not shown).

### Modeling

**Figure 3:**
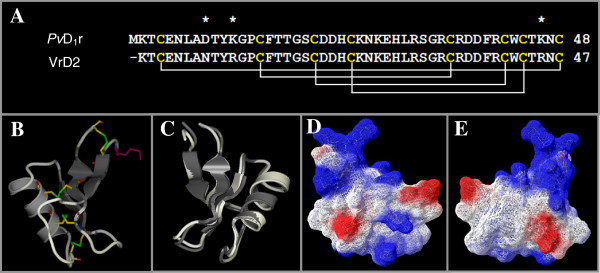
**Modeling. (A)** Alignment of the amino acid sequences of *Pv*D_1_r and VrD2, a defensin isolated from *Vigna radiata*. The lines below the cysteines of VrD2 indicate the disulfide bound formation and paring among the cysteine residues. The three different amino acid residues between the two sequences are indicated by asterisks. **(B)** Three-dimensional structure of *Pv*D_1_r, modeled with the Modeller program and based on the structure of the *V. radiate* defensin VrD2 (pdb CODE 2GL1). Dark gray represents the β-sheets; gray represents the α-helix; and light gray lines represent the unstructured elements. A methionine residue (purple) was added as a requirement for cloning proteins into the pET-32 EK/LIC vector. The four disulfide bridges are shown by the interconnection of the cysteines residues shown in yellow. The sulfur is shown in green, the nitrogen in blue and the oxygen in red. **(C)** Overlap of the three-dimensional structures of *Pv*D_1_r (light gray) and *Vigna radiata* defensin 2 (VrD2) (code pdb 2GL1; in dark gray. **(D and E)** Three-dimensional structure of *Pv*D_1_r with a surface charge; the negative surface charge is shown in red, and the positive surface charge is shown in blue. In E the structure is rotated 180º in relation to D. For an additional explanation of the color usage in this figure, please refer to the web version of the article.

In Figure 3 we present a model of the structure of the recombinant defensin *Pv*D_1_r. First, the amino acid sequence alignment between the *Pv*D_1_r and the selected template, the defensin from *V. radiata* (VrD2; pdb CODE 2GL1), is shown and demonstrates that these sequences differs in only three amino acids residues, as indicated by asterisks in Figure 3A, thereby corresponding to 98% identity. Figure 3B shows that the structure of this peptide is composed of three β-sheets and one α-helix. Four disulfide bridges are present in the structure. Figure 3C shows the overlap of the structures of *Pv*D_1_r and the *Vr*D2 defensin that was used as a model. This overlap shows that there are no significant differences between these structures, and the main differences observed are in the region of unstructured loops. Figure 3D and E shows the positive residues (arginine and lysine) that are exposed on the surface of *Pv*D_1_r. The quality evaluation of the model was performed by analyzing the Ramachandran plots, which revealed a good stereochemistry for the model (result not shown). The RMSD determined for this model was 0.25 Å.

### Effect of *Pv*D_1_r and *Pv*D_1_ on wild type and mutant *C. albicans* growth

**Figure 4:**
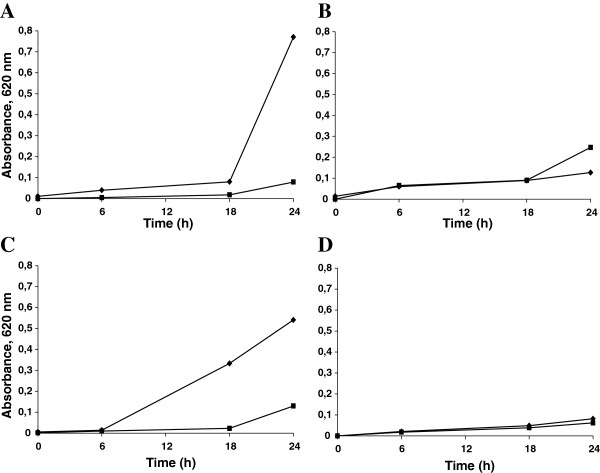
**Effects of *Pv*D**_**1 **_**and *Pv*D**_**1**_**r on the growth of the wild-type and mutant strains of *Candida albicans*.** The absorbance at 620 nm was taken as a measure of *C. albicans* growth. (**A)***C. albicans* wild-type strain; **(B)***C. albicans* mutant strain; (♦) Control; (■) 100 μg.mL^-1^ of *Pv*D_1_; **(C)***C. albicans* wild-type strain; **(D)***C. albicans* mutant strain; (♦) Control; (■) 100 μg.mL^-1^ of *Pv*D_1_r. All of the experiments were performed in triplicate, and the standard errors were omitted for clarity (coefficients of variation were less than 20%).

First, in these assays, we compared the activity of *Pv*D_1_r with regard to *Pv*D_1,_ and we showed that the biological activity of both defensins at 24 h and 100 μg.mL^-1^, which were measured by the growth inhibition of the wild-type *C. albicans* strain, were 75,9 and 89,7%, respectively (Figure 4A and C). This result indicates that the recombinant production of *Pv*D_1_r preserved its biological activity. Comparing the growth of the wild type and the mutant *C. albicans* strains in the absence of the defensins, we observed that the mutant strain grows approximately 84% less than the wild-type *C. albicans* (Figure 4). In the presence of *Pv*D_1_ at 100 μg.mL^-1^, we observed that the mutant strain grew 8.3 and 1.1% more than the control at 6 h and 18 h, respectively. This tendency, observed in the initial hours, increased to 94.4% of additional growth at 24 h with regards to the control (Figure 4B). This result is further analyzed in the Discussion Section. In the presence of *Pv*D_1_r at the same concentration, we observed a good superposition of the growth curves during the time course assayed (Figure 4D). This result indicates that *Pv*D_1_r did not present growth inhibition on the *C. albicans* mutant strain.

**Figure 5:**
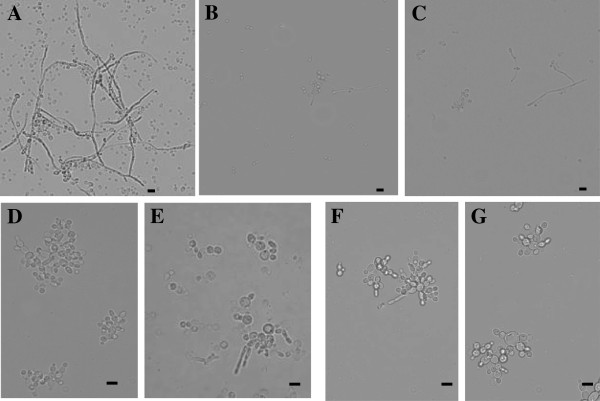
***Candida albicans *cells, wild-type and mutant strains, visualized by light microscopy after the growth inhibition assay. (A)** Wild-type control cells; **(B)** Wild-type cells grown in the presence of *Pv*D_1_; **(C)** Wild-type cells grown in the presence of *Pv*D_1_r; **(D)** Mutant control cells; **(E)** Mutant cells grown in the presence of *Pv*D_1_; **(F** and **G)** Mutant cells grown in the presence of *Pv*D_1_r. Bars = 10 μm.

We further analyzed, through optical microscopy, possible alterations to the morphology of *C. albicans* cells at the end of the growth inhibition assay for both defensins. First, the wild-type and mutant strain presented contrasting growth patterns. The wild type strain presented single ovoid cells as well as long pseudohyphae (Figure 5A). The mutant strains presented aggregated cells with short pseudohyphae and occasionally sparse cells (Figure 5D). With regard to the effect of the defensins on these cells, we noted that in the presence of 100 μg.mL^-1^ of *Pv*D_1_, the wild-type cells were agglomerated and reduced in number and also presented reductions in the size of the hyphae (Figure 5B). The mutant cells, when treated with *Pv*D_1_, showed very similar morphology and growth patterns compared to the control mutant cells (Figure 5D,E). After treatment with 100 μg.mL^-1^ of *Pv*D_1_r, wild-type *C. albicans* cells presented marked reductions in cell number and hyphal size as well as agglomerated cells (Figure 5C). In comparison, the effect of *Pv*D_1_ and *Pv*D_1_r on wild-type cells was visually the same. The mutant cells treated with *Pv*D_1_r presented a very similar morphology and growth pattern when compared to the control mutant cells and the mutant cells treated with *Pv*D_1_ (Figure 5D,F-G).

### Optical microscopy with the fluorescent dyes FITC and DAPI

**Figure 6:**
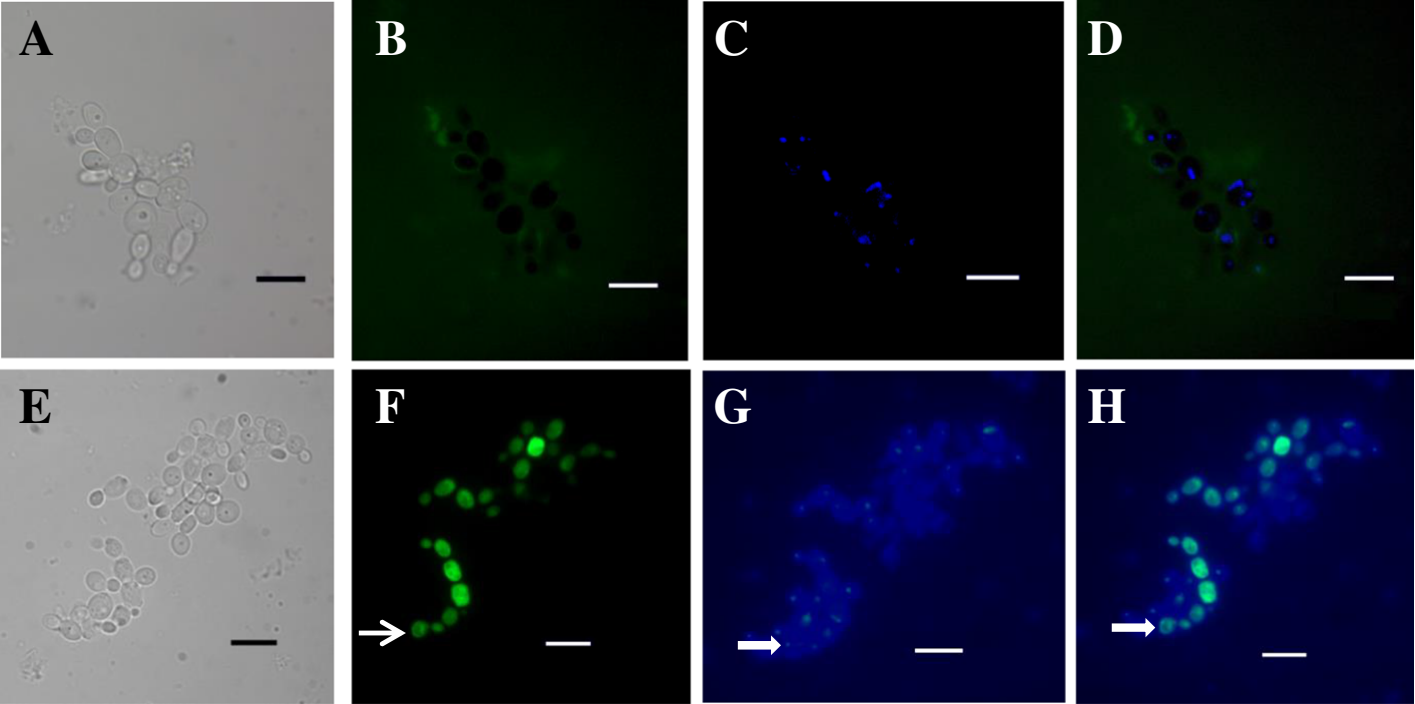
**Fluorescence microscopy of *Candida albicans *cells**. **(A-D)** Mutant cells; **(E-H)** Wild-type cells. Cells were incubated for 24 h with 50 μg.ml ^-1^ FITC-conjugated *Pv*D_1_r (green fluorescence) **(B and F)**. After the incubation period, the nuclei were stained with DAPI (blue fluorescence) **(C and G)**. Light microscopy **(A and E)**. Overlap of the DAPI and FITC images (**D** and **H)**. The open arrow shows the FITC labeling **(F)**; the filled arrows show the DAPI nuclei labeling **(G)** and the colocalization of FITC and DAPI **(H)**. Bars = 10 μm.

To determine the localization of *Pv*D_1_r in the *C. albicans* wild-type and mutant strains*,* 50 μg.mL^-1^ of *Pv*D_1_r was coupled to FITC and used in the growth inhibition assay. Next, the cells were subjected to DAPI labeling. In this assay, we observed that the FITC-conjugated *Pv*D_1_r did not interact with the mutant strain (Figure 6B), while in the wild-type strain, the localization of *Pv*D_1_r was intracellular, as expected due to the presence of the membrane target (Figure 6F). Despite detecting the recombinant defensin *Pv*D_1_r in the intracellular spaces of the wild type cells, this protein colocalize with the nucleus following DAPI staining (Figure 6H).

## Discussion

*Pv*D_1_r was successfully expressed in *E. coli* (Rosetta-gami 2 (DE_3_) pLysS expression strain). This expression system was used to produce recombinant proteins tagged with thioredoxin and six consecutive histidines (Trx and His tags). After induction with IPTG, the bacteria that were transformed with pET-*Pv*D_1_ expressed a fusion protein (Trx-His-*Pv*D_1_r) above the molecular mass marker of 16.9 kDa. This mass corresponded to the molecular size of the expressed Trx-His-*Pv*D_1_r, as determined by SDS-tricine gel electrophoresis (result not shown). Trx-His-*Pv*D_1_r consisted of an approximately 20.4 kDa Trx-His tag and a mature defensin of approximately 6 kDa. It has been previously suggested that Trx, an *E. coli* resident protein, might help to maintain the solubility of a significant fraction of expressed fusion proteins, particularly proteins that would otherwise precipitate entirely as inclusion bodies [22]. This is a basic requirement for the establishment of native disulfide bonds. Additionally, Elmorjani et al. [23] reported that this strategy significantly increased the specific yielded of recombinant fusion proteins. The strategy of expressing *Pv*D_1_r as a fusion protein, however, required a cleavage protocol to allow for the retrieval of the protein of interest from the fusion partner. For this reason, the Trx-His-*Pv*D_1_r fusion protein was submitted to alternating steps of Ni^+^ affinity chromatography and enterokinase cleavage to remove the Trx-His-tags. Figure 2B (Lane 4) shows the electrophoretic profile of the purified recombinant protein. Gel electrophoresis confirmed that the recombinant defensin had the approximately same molecular mass as the defensin that was isolated from the *P. vulgaris s*eeds, according to Games et al. [7], as well as other plant defensins [3,24].

*Pv*D_1_r differed from *Pv*D_1_ by only one amino acid residue: the initial methionine. The presence of this methionine in the N-terminal region of the *Pv*D_1_r was required for protein expression using the pET-32 EK/LIC vector (result not shown).

The modeling of the structure of *Pv*D_1_r was performed with *Vigna radiata* defensin 2 (*Vr*D2) as a template because the primary structure of *Vr*D2, as determined by Lin et al. [25], showed 98% identity with the *P. vulgaris* seed defensin (Figure 3A). Plant defensins are small peptides (45–54 amino acid residues) that have a molecular weight between 5 and 7 kDa and are highly basic and cysteine-rich [26,24]. The structural conformation includes three anti-parallel β sheets and one α-helix, which are stabilized by four disulfide bridges [24,27]. The modeling showed that, like other defensins, the *Pv*D_1_r defensin consisted of three β-sheets and one α-helix, and the three-dimensional structure of the peptide was stabilized by four disulfide bridges. Furthermore, the positive residues (arginine and lysine) that were exposed on the surface of the molecule were important for the antifungal activity of this peptide and have been previously described in other studies [7,9]. The additional methionine did not induce alterations to the modeled structure, as shown by the superimposition of the *Pv*D_1_r with its model template, thus presenting small dissimilarities mainly in the regions of unstructured loops (Figure 3C).

To verify the functionality of *Pv*D_1_r, its effects were compared with those of the natural defensin *Pv*D_1_ in a *C. albicans* growth inhibition assay. A good indication that *Pv*D_1_r was as biologically functional as *Pv*D_1_ was the observation of a soluble band by gel electrophoresis that corresponded to the recombinant Trx-His-*Pv*D_1_r (Figure 2B2). The solubility of the recombinant protein is a good indication that both the folding and post-translation modifications, and in the case of *Pv*D_1_r the formation of the disulfide bounds, were processed correctly [28]. Furthermore, N-terminal amino acid sequence analysis revealed that the overall production process of *Pv*D_1_r, including the cloning of the cDNA into the expression vector, folding, purification and mainly EK cleavage, was successful (result not shown). All of the aforementioned evidence of the functionality of *Pv*D_1_r were further confirmed by its biological activity on the wild-type *C. albicans* strain (Figure 4C). Games et al. [7] previously showed that the natural defensin *Pv*D_1_ could inhibit the growth of *C. albicans* as well as other fungi. Several defensins isolated from various plants have also demonstrated similar inhibition profiles against the growth of microorganisms. Thevissen et al. [29] evaluated the effects of different defensins on the growth of pathogenic yeasts of the genus *Candida*. The plant defensins *Hs*-AFP1 and *Rs*-AFP2 were both able to inhibit the growth of *C. albicans* and *C. krusei*. Another defensin isolated from *Vigna sesquipedalis* seeds showed inhibitory activity against the growth of *Escherichia coli*, *Bacillus megaterium*, *Mycobacterium phlei* and *Proteus vulgaris* bacteria [30]. More recently, Wong et al. [31] showed that a defensin isolated from the *P. vulgaris* cultivar King Bean Pole effectively inhibited the growth of *Mycosphaerella arachidicola*, *Saccharomyces cerevisiae* and *C. albicans* when used in small concentrations. With regards to the inhibition profile of recombinant defensins, several authors have demonstrated that the recombinant counterparts are as active as the natural form [32,33]. However, slight differences between the activities of the natural and recombinant defensins in tests of biological activity have been reported with different reported causes. The recombinant AX2, a defensin isolated from *Beta vulgaris* leaves, showed slight changes in biological activity tests. The authors attributed this difference in activity to the addition of an extra amino acid at the N-terminal region as required by the cloning approach [34]. The recombinant *Vr*CRP, a defensin isolated from *Vigna radiate*, presented toxicity to *E. coli* and could only be expressed in this system when bound to a truncated signal peptide and a chitin binding domain tag [35]. Another peptide, called brazzein, isolated from *Pentadiplandra brazzeana*, presented the same scaffold of the plant defensins and stimulated an intense sweet taste in primates, however, when expressed recombinantly, it presented low intensity sweetness in a sensory analysis. The authors explained that the low intensity sweetness occurred due to incorrect folding in the recombinant system [36].

After confirmation that *Pv*D_1_r was biologically active, we evaluated the activity of *Pv*D_1_ and *Pv*D_1_r against a ΔGCS strain of *C. albicans*. This mutant strain lacks the gene for the enzyme GCS, a protein that is required for the formation of the sphingolipid glucosylceramide (GluCer) on *C. albicans*. The superposition of the growth curves of the control and the test indicated that *Pv*D_1_ and *Pv*D_1_r did not present toxic effects on this strain. This result was expected because the supposition of the membrane target of this defensin is the sphingolipid GluCer, which is absent in the membrane of this mutant strain. Without its membrane target, *Pv*D_1_r did not interact with the membrane and, consequently, did not trigger toxic effects. Other fungal species that were null for the sphingolipid gene presented the same effect when incubated with the particular plant defensins that use the sphingolipids as a membrane target [37,10].

Light microscopy analysis showed morphological alterations in the wild-type strain of *C. albicans* treated with 100 μg/mL^-1^ of both *Pv*D_1_ and *Pv*D_1_r (Figure 5). Several plant defensins were shown to induce morphological alterations in fungal cells [38,39]. These alterations include cellular agglomeration, cellular elongation, hyphae hyper-branching and mainly biomass reduction. Some authors also reported that these effects vary in intensity from mild to severe and also vary in regard to the combination of plant defensin and fungal species [39-43]. With regards to the effect of *Pv*D_1_ and *Pv*D_1_r on the mutant yeast strain, no significant difference was observed (Figure 4). This result was expected because this yeast strain lacks the sphingolipid GluCer caused by the absence of the GCS enzyme that synthesizes the sphingolipid. Our results, as shown in Figure 4, demonstrate that cells from the mutant strain appear to have their growth stimulated in the presence of *Pv*D_1_ but not in the presence of *Pv*D_1_r. Microscopy analysis clearly demonstrated aberrant growth of the mutant strain compared with the control (Figure 5A and D).We believe that this aberrant growth pattern, as exhibited by the mutant strain, may increase or interfere with the optical densities that were measured during the growth inhibition assay and may have caused an apparent absence of inhibitory activity (Figure 4B). Morphological alterations, such as cell aggregation and pseudohyphae formation, which are induced by treatment with AMPs, can cause an anomalous optical density in the growth inhibition assay, and this parameter can be used as an indicator of fungal growth, as reported in AMP *Cc*-LTP1 [44]. The inhibition can be confirmed in these cases by observing the culture in the presence of the AMP at the end of the growth inhibition assay by optical microscopy (Figure 5).

The biological function of *Pv*D_1_r was also confirmed by fluorescence microscopy analysis. In this analysis, we show that *Pv*D_1_r did not interact with the mutant strain lacking the GCS gene, and the labeled proteins were only observed outside of the cell (Figure 6B). In sharp contrast, this labeling was observed inside the wild-type strain, indicating that the *Pv*D_1_r interacts with the membrane target GluCer and enters the cell (Figure 6F). The incorporation of plant defensins into the fungal cytoplasm was observed for *Na*D1 (isolated from *Nicotiana alata*), which was able to enter the *F. oxysporum f. sp. vasinfectum* hyphae but was not observed in the nuclei [45]. In contrast, Lobo et al. [46] showed that the defensin isolated from *Pisum sativum*, *Ps*D1 could in fact have a nuclear target. This study and other related studies suggested that the antifungal activities of plant defensins are not restricted to the plasma membrane of fungi, as the defensins can enter the cells and target different intracellular compartments.

## Conclusion

We cloned the *Pv*D_1_ coding sequence into a pET-32 EK/LIC vector, and this construct was used to transform the *E. coli* expression strain Rosetta Gami 2 (DE_3_) pLysS. The expression and purification were successful. The presence of a soluble band above the 16.9 kDa marker was a good indication of the correct folding and formation of disulfide bounds in the recombinant protein. The biological functionality of *Pv*D_1_r was confirmed by comparing its activity to the activity of *Pv*D_1_ against wild-type *C. albicans*. Both peptides presented very similar biological activity, with a growth inhibition of 75,9 and 89,7%, respectively. These results were corroborated by microscopic analysis of wild-type *C. albicans* treated with *Pv*D_1_r and *Pv*D_1_. The cells treated with both peptides were agglomerated and reduced in number and also had smaller sized hyphae. To discover the target of the *Pv*D_1_r in *C. albicans,* we used a mutant strain lacks the gene glucosylceramide synthase (GCS1), which synthesizes the sphingolipid GluCer. Our results with this *C. albicans* mutant strain indicated that this strain is resistant to *Pv*D_1_ and *Pv*D_1_r compared with the wild-type strain. This conclusion was reached due to the absence of *Pv*D_1_ and *Pv*D_1_r activity in the growth inhibition assay, the observation that the morphology of the mutant strain was not affected and the finding that *Pv*D_1_r did not enter the mutant strain cell. This final result not only demonstrates that *Pv*D_1_r requires GluCer at the membrane of *C. albicans* as a target to trigger it toxic effects but also that the defensin from *P. vulgaris* may have an intracellular target as well.

## Abbreviations

AMP: Antimicrobial peptides plant; Das: Antisense primer; Ds: Sense primer; DAPI: 4’,6-diamidino-2-phenylindole dihydrochloride; EDTA: Ethylenediamine tetraacetic acid; EK: Recombinant bovine enterokinase; FITC: Fluorescein isothiocyanate; GluCer: Glucosylceramide; His: Histidines; HPLC: High-performance liquid chromatography; IPTG: Isopropylthio-β-galactoside; LIC: Ligation-independent cloning; PCR: Polymerase chain reaction; PvD1: *Phaseolus vulgaris* defensin one; PvD1r: Recombinant defensin; RMSD: Root-mean-square deviation; RT-PCR: Real-time polymerase chain reaction; TFA: Trifluoroacetic acid; S: Glutathione; Trx: Thioredoxin; (Δ)GCS1: Deficient on glucosylceramide synthase (GCS) enzyme.

## Competing interests

The author(s) declare that they have no competing interests.

## Authors’ contributions

EOM carried out the all research and analysis and drafted the manuscript., ISS carried out the molecular biology experiments, AOC carried out in data analysis and drafted the manuscript, LSS carried out the purification of the natural *Pv*D_1_, GAS-F participated with the molecular biology experiments, VVN carried out the modeling experiments, OLTM carried out the sequencing of the *Pv*D_1_r, UZ carried out the FITC labeling of *Pv*D_1_r. VMG participated in its design and coordination and helped to draft the manuscript. All authors read and approved the final manuscript.
